# Older surgical patients’ preferences for follow-up care after hospital discharge: A multi-method qualitative study into their underlying needs

**DOI:** 10.1016/j.ijnsa.2025.100394

**Published:** 2025-07-29

**Authors:** Aafke J de Groot, Marike E de Boer, Elizabeth M Wattel, Cees MPM Hertogh, Marja FIA Depla

**Affiliations:** a Department of Medicine for Older People, Amsterdam UMC, Vrije Universiteit Amsterdam, De Boelelaan 1117, 1081 HV Amsterdam, the Netherlands; b Amsterdam Public Health Research Institute, Aging & Later Life, de Boelelaan 1117, 1081 HV Amsterdam, the Netherlands

**Keywords:** Aged, Patient discharge, Patient preference, Qualitative research, Transitional care

## Abstract

**Background:**

Almost 30 % of older patients suffer from functional decline during hospital stay which often makes follow-up care decisions necessary. However, little is known about the topics that are relevant to these patients and their families to address in discharge conversations.

**Objective:**

This study aims to provide insight into the perspective of older surgical patients on follow-up care by exploring their considerations expressed throughout the process of discharge planning.

**Design:**

A multi-method qualitative study

**Participants:**

Participants were older patients from a surgical ward who anticipated to require follow-up care after discharge.

**Methods:**

Data collection included: 1) interviews with patients; 2) informal conversations with family members and professionals; 3) non-participant observations during medical rounds and bedside conversations; 4) extraction from medical records. In all data sources, the focus was on capturing the patients’ considerations with regard to follow-up care. We employed inductive thematic analysis to identify needs that underlie patients’ preferences for follow-up care.

**Results:**

Twelve older surgical patients with complex or delayed discharge were followed. Their considerations with regard to follow-up care revealed five underlying needs: 1. Safety (adequate care), 2. Familiarity (trusted people and surrounding), 3. Independence (active and autonomous living), 4. Continuity (resume previous life) and 5. Relief (ending endurance). Most participants had more than one need.

**Conclusions:**

Older surgical patients’ perspective on follow-up care can be captured by five underlying needs. Gaining insight into these needs contributes to a better understanding of patients’ preferences for follow-up care. We therefore recommend exploring these needs in older surgical patients, as well as identifying potential similar or additional needs for other subgroups of older hospital patients, as an important step toward personalized decision-making in transitional care.

## Introduction

Almost 30 % of older patients suffer from functional decline during hospital stay, causing temporary or structural impairments in activities of daily living. As these impairments could hinder immediate independent living after discharge, the end of a hospital episode can mark the beginning of personal adjustment to home care support or imply admission to a care facility for further recovery (Gabrielsson-Järhult and Nilsen, 2016; Galvin et al., 2017; Sorkin et al., 2018). The episode after hospital discharge may thus mark important transitions in the lives of older patients.

To support older patients during these transitions, extensive research has been done on their discharge process from hospital. Initially, studies predominantly focused on (personalized) discharge planning as an intervention to prevent adverse outcomes of care. Cohort studies found mixed results: Braet and colleagues reported positive effects on readmission rates, whereas a Cochrane review found little evidence on length of hospital stay and readmission rates comparing personalized with standard transitional care (Braet et al., 2016; Gonçalves-Bradley et al., 2022). To improve transitional care, interventions are recommended that start well before discharge and include communication with the family (Bauer et al., 2009).

Other hospital discharge studies have also addressed the satisfaction and involvement of older patients and their family caregivers in the hospital discharge process. Studies have focused on topics such as readiness for discharge, discharge communication and shared decision making (Fiore et al., 2012; Galvin et al., 2017; Mabire et al., 2016; Hesselink et al., 2012; Graham et al., 2024; Hoefel et al., 2020; Manhas et al., 2020; Burke et al., 2018). In short, recommendations from these studies suggest that patient and caregiver should be equal and well-informed partners in conversations on hospital discharge planning (Mennuni et al., 2017).

These recommendations are in line with the view that shared agreement applies to follow-up care decisions as well as to other treatment decisions (Ottenvall Hammar et al., 2014). This principle is affirmed when discharge conversations openly address all topics of discharge and follow-up care relevant to patients and their families. However, little is known about the topics that are relevant to older hospital patients for whom follow-up care is unavoidable. Which topics should these specific discharge conversations openly address?

We do know that in a discharge process, preferably, time should be allocated to explore the patients’ preferences for further treatment by asking questions that reveal their perspective on their own recovery. Issues that may arise are how the period after leaving the hospital is perceived and the extent to which the patient feels ready for discharge (Gadbois et al., 2019; Heppenstall et al., 2014; Gadbois et al., 2017).

In addition to existing knowledge, we want to further explore the older patients’ perspective on follow-up care after hospital discharge. We have chosen to focus our study on the subgroup of older hospital patients who had undergone major surgery, hereafter referred to as ‘older surgical patients’. As these patient group require a wide range of post-acute care needs, including specialist homecare, rehabilitation oriented post-acute care or palliative care, they also may provide information on the perspective of older hospital patients in general.

Hence, this study aims to gain a comprehensive understanding of older surgical patients’ preferences for follow-up care by exploring the considerations they express throughout the process of discharge planning. What are their wishes, uncertainties and concerns regarding follow-up home care and (temporary) residential care? Which needs may account for their preferences for a special type of follow-up care? Exploring these considerations among older surgical patients is expected to contribute to decision-making in transitional care for older hospital patients in general.

## Materials and methods

### Design

This study used a qualitative, multi-method design, that incorporated interviews, observations, and the extraction of medical records. It involved four months of fieldwork studying older surgical patients’ considerations during the process of hospital discharge planning. The multi-method approach was chosen to gather information from as many sources as possible on what patients considered throughout this process.

The authors formed the research team with qualitative expertise (B, C, E), qualitative research training (A), and medical or geriatric rehabilitation expertise (A, D, E).

### Setting

Data were collected on a 24-bed surgical ward in a tertiary hospital, treating patients with inflammatory bowel disease, colorectal tumors or neuro-endocrine oncological disease. The average patient turnover on this ward was over five per day. Post-operative care typically covered 3–5 days of in-hospital recovery. When discharge home was deemed unsafe, a liaison nurse was involved to arrange follow-up care. The professional team consisted of surgeons (head practitioners), residents, specialized and junior nurses, allied health care professionals, and medical consultants.

Several aspects of the workflow were relevant in assessing patients’ considerations in discharge planning. Nurses were assigned to new patients daily, based on individual case complexity, balancing the workload across the nursing team. This may have limited continuity of care and opportunities to develop familiarity with patients. In addition, reports on patients’ considerations related to discharge and follow-up care were documented in their medical records. Lastly, transitional care and discharge discussions were often conducted on short notice, typically without family present.

### Sampling

We used purposeful sampling with a focus on older patients that were expected to require some sort of follow-up care, as we strived for information rich cases in terms of hospital discharge considerations. Therefore, criteria for inclusion were: older patients (>55 years) for whom 1) discharge planning had started, and 2) discharge to follow-up home care or temporary residential care was foreseen. Patients were not eligible for inclusion when 1) they were too weakened to provide written consent or participate in an interview, 2) decisional incapacity prevented them from participating, or 3) ‘not speaking XXXX or English’ would hinder participation in the study. Based on these criteria, eligibility for the study was assessed by an attending physician or resident, in consultation with the researcher (A) conducting the fieldwork. She was present during rounds three days a week to assist with recruitment. To keep the data collection manageable, a maximum of 2 patients were followed at the same time. When a patient was deemed eligible, the attending physician introduced the study during a bedside consultation and asked permission for the researcher to start the informed consent procedure.

Of all patients approached by the researcher only two refused to participate, with no further explanation beyond their unwillingness. Another six patients consented to participate but could not be included due to: (1) the development of delusional complaints, (2) change in severity of illness preventing further participation, (3) withdrawal of initial informed consent and (4) transfer to another hospital ward. One participant (P4) was adamant in referring us to his partner to be interviewed because XXX was not his first language and engaging in a longer conversation would be exhausting.

Twelve patients were included in the study, six women and six men. Their mean age was 70 (55–82) years. Ten participants had undergone abdominal surgery and two had been surgically treated for a neuro-endocrine disease. Their hospital stay varied from 6 days to 67 days (see Table 1). Three participants were admitted more than once.

**Table 1:** Characteristics of included patients.

	Age	Sex	Living situation	Admission	Surgical procedure	Days in hospital	Follow-up care
P1	70–75	F	with partner	acute	abdominal	6,7,9^1^	home care
P2	55–60	F	living alone	acute	abdominal	18	geriatric rehabilitation
P3	55–60	M	with family	sub-acute^2^	endocrine	19	home care
P4	55–60	M	with partner	acute	abdominal	58	geriatric rehabilitation
P5	70–75	F	with partner	acute	abdominal	67	geriatric rehabilitation
P6	70–75	F	with partner	elective	abdominal	14	home care
P7	65–70	M	living alone	elective	abdominal	8	home care
P8	60–65	F	living alone	elective	abdominal	25	home care
P9	70–75	F	LTC-facility^3^	acute	abdominal	23,16^2^	long-term care
P10	80–85	M	living alone	sub-acute^2^	endocrine	7	home care
P11	80–85	M	with partner	elective	abdominal	63	geriatric rehabilitation
P12	75–80	M	with partner	elective	abdominal	8,6^2^	geriatric rehabilitation

1 Readmitted after initial discharge home.

2 Priority admission: within days.

3 LTC=Long term care.

### Data collection

Data collection took place from September to December 2021, and included: 1) interviews with patients; 2) informal conversations with family members and professionals; 3) non-participant observations during medical rounds and bedside conversations; 4) extraction from medical records. In all data sources, the focus was on capturing the patients’ considerations with regard to follow-up care. To the extent that family members and health professionals were used as sources, these represented indirectly expressed considerations.

### Interviews

Formal interviews were conducted with patients, guided by a topic list exploring their considerations regarding follow-up care (see suppl. A). These interviews lasted between 15 and 20 minutes, were audiotaped, and transcribed verbatim. In the two cases where audiotaping was declined, notes were taken and the content was written up immediately after the interview.

*Informal conversations with family members and professionals*

Informal conversations with family members and professionals were conducted to understand the considerations patients expressed to them during the process of discharge planning. To increase the likelihood of speaking with them, we opted for unscheduled ‘go-along’ interviews. These conversations were guided by open questions concerning the patients’ view on follow-up care. They lasted between 5–20 minutes and were not audiotaped; a report was written immediately after each conversation.

*Non-participant observations of medical rounds and bedside conversations*

Observational data were collected during daily medical rounds, when discharge plans for included patient were discussed between nursing and medical staff. Additional observations focused on bedside conversations on discharge and follow-up care between the attending physician and the patient. These conversations lasted between three and ten minutes, with a family member occasionally present. Field notes of both types of observations were written within the hour and summarized into reports on a regular basis. As a non-participant observer the researcher had minimal influence on the data collected.

### Medical records

Medical records were screened for professionals’ notes referring to the patients’ perspectives on discharge planning and follow-up care, and relevant data were copy-pasted. The professionals whose notes were included were nurses, physicians, allied healthcare professional, and medical consultants.

An overview of the data collection for each case is provided in Suppl. B.

### Data analysis

We combined the data from interviews, informal conversations, observations, and medical records into a single chronological dataset per patient. Since the data collection method did not significantly impact the analysis, we treated the datasets as integrated, without focusing on individual sources. In other words, each patient’s case history was formed with input from the patient, family, and professionals.

To analyze these individual case histories, we used thematic content analysis, which allowed us to identify recurring themes and underlying patterns inductively (Braun and Clarke, 2006). Firstly, two researchers (A, B) familiarized themselves with the data by reading and rereading the case histories. Data reflecting the participants’ perspective were independently selected and coded (A, B). Next, based on the open coding of the case histories, data was taken to a higher level of abstraction by writing case-specific memos on the participant’s considerations on follow-up care (Birks et al., 2008). Two examples of such memos are provided in suppl. C. In the third step, coded considerations and memos were compared and discussed between cases (A, B, C), leading to the preliminary identification of themes related to the needs underlying participants’ considerations. Finally, the results were discussed within the research team leading to consensus on merging the themes into main needs. All data analysis steps were discussed with the research team (A, B, C, D, E). An overview of codes, themes and main themes emerging from the individual case histories is provided in suppl. C.

In the reporting of the study we adhered to the COREQ guideline (suppl. D)

### Ethics

The medical ethics review committee (METC) of the participating university medical center approved the study protocol (METC file no. 2020.302). Prior to data collection written informed consent was obtained from the nursing staff and the permanent medical team of the ward. All other participating professionals, as well as the included patients and their family members, provided written informed consent when they became involved in the data collection.

## Results

Analysis of participants’ considerations concerning follow-up care identified five underlying needs: 1. Safety, 2. Familiarity, 3. Independence, 4. Continuity, and 5. Relief. In the data presented, we specify the participant to whom the considerations pertain, as well as the source of the considerations (interview with patient, conversation with family or professional, observation of medical round or bedside conversation, report in medical record).

### Safety

All participants had recently undergone major surgery. When thinking of hospital discharge and follow-up care, a first important need was to feel safe and be strong enough despite their vulnerable condition. Participants expressed that they wanted to go home if they had recovered well enough and when proper, secure and adequate medical care was arranged at home so that no mishaps or complications would occur. When prompted, participants mentioned to worry about nursing aids for their colostomy, tube feeding arrangements, the proper wound care, their medication and the follow-up surgical treatment.

*‘Like this I cannot leave the hospital. The doctors talk about going home, but first of all my bowels (intestines) must be in order. Me and my husband we can take care of it [the colostomy], though the pouching system they use here is different.’ [P6, interview with patient]*

*‘She insisted constantly that we restart her old medication and take care of the old fistula.’ [P6, observation medical round].*

*‘She needed reassurance and explanation more than once.’ [P6, nurse report]*

*‘She considers that having time for recuperation would be beneficent for her, but on the other hand she would prefer to go home would that be possible. The vacuum-assisted closure (VAC)-pump treatment, however, every third day and very painful, urges her to prefer in-patient post-acute care (in a nursing home)’ [P2, nurse report]*

*‘I am very worried about the endo-procedure he [P4] needs. He would have to be transported twice a week from the rehabilitation facility back to the hospital. He is not capable of doing that, it would be too much of a burden.’ [P4, conversation with partner]*

Some participants knew from experience how helpless and desperate one feels when no help is at hand in an emergency situation. They didn’t want to go through that again and preferred a temporary stay in a skilled nursing facility.

*‘I will absolutely not go directly home from here; what happened at home earlier scared us. I meant to get up, then suddenly I became unwell and ended up on the floor! My wife could not help me, she has a heart condition. We telephoned the family doctor and he had me re-admitted at once. Now I need to have my intestines functioning before I go home. I need physiotherapy and a fortnight stay in a nursing home, to rest. In a hospital there is no chance of resting. My wife said: “Of course I want you home, but you need recovery first”.’ [P12, interview with patient]*

*‘Eleven years ago I went through the same experience. Six months it took me then, slowly recovering at home, assisted by home care workers. At eleven o’clock in the evening the last of them left and I was alone for the night. Well, back than I was 10 years younger, now it feels quite different. I have reconsidered, I would prefer to go home straight, but then I will be alone at night.’ [P8, interview with patient]*

Being in a nursing home was not entirely reassuring in itself. One participant was afraid that prompt availability of care was not guaranteed when she would suddenly need assistance there.

*‘How do you feel about going to a nursing home?’ ’It scares me’ ‘Does it scare you?’ ‘Yes, I am afraid that I am left waiting. That they are not attentive so that it takes a long time waiting for help. That they don’t notice my call for help.’ [P8, interview with patient]*

### Familiarity (‘familiar-ness’)

Pondering on the end of hospital stay, a need for familiar surroundings and familiar people helping them was expressed as well. In some cases this was a strong desire to be with their own, familiar and trusted people once again. One participant consented with any given post-acute care planning as long as he could leave without delay and go home, in his case a farm where he had lived all his life.

*‘He has had a tremendous outburst, he cannot stand being here for so long and he misses his family very much.’ [P3, observation medical round]*

*He is frustrated that he was told that he could go home, but then the next day the decision was withdrawn and this happened more than once. His family cannot visit often, this adds to his feelings of frustration and anger. But in the end he admitted that discharge would also have to be safe’. [P3, nurse report].*

The need for familiarity of another participant was expressed in her wish to be supported only by her husband and daughters after discharge. For her, formal home help was acceptable only in case her family wouldn’t have the expertise to nurse her.

*‘I very much long to go home!’ ‘What will you need to recover at home and regain strength?’ ’My husband will help me, he and I will take walks together. He will take care of me when I get home. He makes a delicious soup and together we will manage.’… ‘Do you need help from homecare workers?’ ‘Absolutely not! Me and my husband, we can easily manage together, the wound is simple this time’. [P6, interview with patient]*

In contrast the need for familiarity can also lead to a wish to postpone discharge. After a three week stay in intensive care followed by an even longer stay at the ward, one participant felt that the hospital department and team had become so familiar that he preferred to stay there, instead of being transferred to a new environment.

*‘The departure and goodbye from the team is very emotional, he will miss us all very much. Going to a new place is stressful. He and his partner feel defeated, it is all going so fast.’ [P4, nurse report]*

### Independence

A third category of considerations showed the need and desire to be independent, to experience freedom and autonomy and to spend the day according to one’s own choice and go wherever wanted. When confronted with hospital discharge, these participants expressed their wish to be independent again, to be free and on their own. In these considerations, concerns about fear to have lost the necessary (physical) independence were uttered as well.

One participant feared to be sincerely patronized in hospital and had postponed a necessary surgical treatment for years. Once in hospital, he wanted to be liberated from the unwanted interference of others in his lifestyle as soon as possible and left the hospital without waiting for prescriptions of medication.

*‘Yes, after this weekend, there is a chance I can go home. I am absolutely ready for that! I dreaded the dependency in being a hospital patient. I cherish my privacy and I like being on my own, I want to be able to smoke again [shows Nicotine bandage]’. [P9, interview with patient]*

The need for freedom and independence could also be an expression of a deeply felt desire to be active, to be up and about. For these participants a physical condition allowing for independent mobility was pivotal whereas to them the prospect of needing nursing home care would imply living a dependent and passive life.

*‘For me, this diagnosis [a malignant tumor] is bad news; I fear going home towards a future with this.. maybe I must realize the facts: my time is over. I used to work with quite a few older colleagues and they are all in residential care homes now, passively sitting and nothing ever happens.’ [P10, interview with patient]*

*‘I know for certain that he will not want to go to a nursing home. That is not adequate for him, that really does not suit him, mentally spoken’. [P11, conversation with nurse]*

*‘They ask me whether I can stand it, but they don’t ask him! It is such a hardship for him, he is such an active person and now he has been constrained to bed for seven weeks’. [P11, conversation with partner]*

The need to live independently was an influential motive in considerations of participants. Not being able to live an independent life again would make their life unbearable. One participant eventually complied with a temporary stay in a nursing home, after an earlier statement that he would prefer death if it came to that.

*‘At the family meeting he was very clear, he wanted to go home or die; not go to a nursing home’. [P11, conversation with physician]*

### Continuity

A fourth need underlying participants’ considerations on discharge was to experience continuity. We appreciated this as a strong wish to go on with their previous lives, the usual and normal life, as it was lived before. For these participants continuity also involved the idea of themselves being the same person as before. Participants expressed this desire and expected to bounce back into that former life, with all the capabilities they had before. A certain degree of denial of the consequences of one’s illness may be heard in this need.

One participant assumed that he could take up his usual family tasks after a short period of recuperation.

*‘I am the cook in the family. For now they have to do the cooking themselves and I hear them about ordering pizza’s! But they also tried to make a home-cooked meal, so I see some progress. Normally, I also do the cleaning in the house, seeing those tiles shining again makes me content. My wife cannot do these things.’ [P12, interview with patient]*

Another participant, who was readmitted multiple times, struggled with the notion that her life could not return to normal anymore. She lived with a husband diagnosed with Alzheimer’s Disease. When asked about the upcoming hospital discharge she expressed grief, in that moment realizing that she was losing her life as it had always been. She also expressed frustration towards her husband and family for lack of understanding what she needed them to do. She felt that they were not offering help in the way she needed it to experience the usual way of things again.

*‘What do you need to recover at home?’ ‘Well, eh, that I don’t have to do everything, ..[hesitates]..because, you know, he is not going to do the shopping… Yes, imagine you come home from hospital and there is no food in the house, nothing…’ [P1, interview with patient]*

*‘You told me your children want to help you?’ ‘Yes, but they want to do the shopping all in once, during the weekend. I would want to get out of the house regularly. Take a walk and have them do what needs to be done.’ [P1, interview with patient]*

This participant did not succeed in compromising between available help and her own need for continuity. She could not find a match with the available help and left the hospital without arrangements for care.

### Relief

In the participants’ considerations on discharge and follow-up care another underlying need was expressed as well: a deeply felt longing that all medical treatment would come to an end. This resulted in a dismissive or downright negative attitude towards any form of follow-up care including rehabilitation. Participants driven by this need made it clear, in word and gesture, that it has been enough, that they wanted no treatment anymore.

One participant had a history of cancer treatment and was now diagnosed with a malignant tumor again. During his long post-operative trajectory with grave complications, he grew passive, silent and repellent towards further treatment of any kind.

*‘I want no treatment anymore.’ [P11, observation bedside conversation]*

*‘It is unbearable, I never wanted this. My situation really is hopeless. Now they even gave me oxygen, I never had that before’. [P11, interview with patient]*

*‘Beforehand he had refused surgery, but since his physical performance was really good they could operate on him and he then complied. Since than he has expressed several times that all this is too much for him.’ [P11, consultant report]*

Another participant with a long medical history, mobility impairments and chronic pain initially refused all medical treatment after an acute readmission from home.

*‘What would be her own wish?’ ‘She herself wants nothing’. ‘Nothing?’ ’She really wants nothing. She wants to smoke, she wants no fussing, no hassle, nothing’. [P5, conversation with physician]*

Her family, who felt differently, succeeded in convincing her step by step to accept and comply to treatment and to a transfer to geriatric rehabilitation.

## Discussion

To gain a comprehensive understanding of older surgical patients’ preferences for follow-up care this study explored the wishes, uncertainties and concerns regarding follow-up care that these patients expressed throughout the process of discharge planning. From their considerations, including those indirectly voiced through family members or professionals, we were able to identify five underlying needs: safety (adequate care), familiarity (trusted people and surrounding), independence (active and autonomous living), continuity (resume previous life), and relief (ending endurance). Most participants expressed more than one need in their considerations.

The underlying needs we identified may elucidate patients' preferences for follow-up care. For instance, a strong rejection of inpatient follow-up care may reflect a significant need for familiarity for one patient, while the same rejection may be driven by an underlying need for independence for another. Similarly, a need for independence may also motivate a patient's preference for a rehabilitation trajectory. As such, the associations between needs and preferences are not universal. However, gaining insight in the underlying needs of patients may enhance understanding of their individual preferences for follow-up care.

All participants had recently undergone major surgery. Consequently, when considering hospital discharge, an important need was to feel safe and be strong enough to leave the hospital. Regarding this need for safety there appears to be no difference between the patient’s perspective and the medical perspective. Patients as well as caretakers were concerned about wound healing, infections, and other potential urgent problems. Therefore, the patients’ considerations regarding safety seem to align with the medical discourse on ‘readiness for discharge after colorectal surgery’, which includes guidelines concerning pain management, oral intake, lower bowel functioning and absence of complications (Fiore et al., 2012; Kelly et al., 2016). However, this does not exclude the possibility of differing opinions regarding the safety of an individual patient. In our study, we observed patients who were more anxious about their recovery than the professionals deemed necessary. This finding supports Hyslops’ recommendation to use the patients' own perspective on risks as a starting point to reach person-centered decisions (Hyslop, 2020).

The need familiarity often underlies a strong preference to return to family and loved ones. This preference may be attributed not only to a strong bond with home but also to the significant difference between the hospital setting and one's own home. In an interview study on the complex care transitions of colorectal patients the ‘wish to return home’ was identified as one of the overarching themes as well (González et al., 2017). According to the authors, dissatisfaction and tensions concerning hospital stay were strongly related to the contrasts patients experience between the hospital with its strict system of procedures and medical rules and the familiarness of their own home. In our study, however, we also observed a patient (P4) who, after a lengthy hospital stay, had developed a strong attachment to the staff and routines of the ward, and therefore did not want to move to a different setting. The need for familiarity could thus manifest as a form of hospitalization as well, wherein the contrast between life in the hospital and the outside world is fading.

For older hospital patients arrangements of follow-up care after discharge may imply a transition to care dependency. The underlying needs for independence and continuity seem to most accurately reflect the patient’s concerns about this issue. The need for independence for instance could be an expression of a deeply felt desire to be active, to be up and about, and a corresponding strong rejection of nursing home care, as we saw in one of our participants (P10). As such, independence includes what elsewhere is described as the need to be one’s own master, signifying a want for autonomy, self-rule and freedom that transcends physical independence as a need (Salisbury, 2019). In the need for continuity, there may even be a certain degree of denial of the consequences of one’s illness. In our study, some participants expected to bounce back into one’s former life, with all the capabilities they had before. They appeared to accept only follow-up care that could help achieve this goal. Consequently, a significant discrepancy between the patient’s and the professional’s perspectives can emerge in discussions about the need for independence and continuity. In one of such cases the patient was discharged without any arrangements being made; none of the proposed options provided the desired continuity (P1).

Relief, the strong wish that all medical treatment would come to an end, is yet another underlying need that implies a rejection of follow-up care. Simultaneously, there appears to be more at stake, as this need for relief of the burden of all medical care may imply that the patient is accepting the end of his life. This may explain that, despite our finding of these wishes being noted in reports, it seemed difficult for doctors and family members to address them. The patients in our study were persuaded to rather take a next step in their treatment and care process. These observations align with the reluctant acceptance of transition to follow-up care, found in a study on patient and caregiver experiences with delayed discharge from a hospital setting (Everall et al., 2019).

Based on a multinational study of a similar population, adult patients who had undergone abdominal surgery, it appears that no needs were overlooked. Their answers to ‘what recovery essentially meant to them’ can be easily linked to the needs found in our study: ‘resolution of symptoms’ (corresponding to the need for safety), ‘returning to habits and routines’ (corresponding to the need for continuity) and ‘regaining independence’ (corresponding to the need for independence) (Rajabiyazdi et al., 2021). Since the participants of this study had already been discharged home, the need for familiarity, as found in our study, was no longer a concern for them.

All five underlying needs were identified partly through notes in the medical records, primarily reports of nurses and consultants, as indicated by their quotes in the results section. This demonstrates that conversations with older surgical patients regarding discharge and follow-up care did certainly take place and extended beyond the practical questions of 'who, what, where, and when'. However, these conversations were held rather casually and their reports were scattered throughout the medical record. To get a more comprehensive picture of older surgical patients’ needs concerning follow-up care, the concepts of safety, familiarity, independence, continuity and relief that were identified in this study may be helpful. They might be used as a framework to listen to patients’ underlying needs and to invite them to discuss potential needs that they do not bring up on their own initiative. In doing so all team members could report their observations and interpretations of patients’ preferences in a more systematic way. To amplify the older patient’s voice amidst ‘the noise’ of a hospital ward, a team approach is therefore needed.

This study was conducted in a surgical department for patients with colorectal or inflammatory bowel disease or with neuro-endocrine oncological disease. As expected this ward generated rich data about the patients’ perspective on follow-up care. The patients were not only referred to geriatric rehabilitation, an obvious discharge destination in trauma, orthopedic or neurological wards, but also to specialized homecare, other inpatient supportive care and even palliative care. As a result, we were able to identify a relatively broad range of underlying needs with a relatively small number of participants. We therefore find it plausible that the predominantly abdominal surgical patients in our study are representative of the broader population of older surgical patients.

However, as our study focused on older surgical patients, it is important to recognize that the identified needs may not be fully transferable to older hospital patients in general. For example, the specific care needs related to wound healing, bowel functioning, and nutrition in our population may have contributed to the surgical-specific underlying need 'safety.' Nevertheless, safety - particularly in terms of the prompt availability of care - may still be a relevant factor in discharge conversations for other older patients. Conversely, research on different subpopulations may reveal needs that are less relevant for surgical patients. Therefore, future research is needed to assess whether our findings are transferable to other subgroups of older hospital patients, such as those with trauma or neurological conditions.

A major strength of this study is that the research was conducted prospectively and on location. Data were collected ‘in the moment’ of the decision making process and without intervening in the ‘real world character’ of the surgical ward. The topics discussed in the interviews were specific and realistic as patients were waiting for hospital discharge. Furthermore, the different methods of data collection allowed for capturing a comprehensive range of patients’ considerations throughout the discharge planning process, including those expressed when the researcher was not present.

However, we encountered limitations as well. Observation of bed-site visits were limited due to a combination of unplannable workflow on the ward and limited availability of the researcher. As a result, data collection gradually shifted from observational methods to interviews and informal conversations with patients and staff. More observations of bed-side conversations on discharge and transitional care might have shed more light on the arguments that patients, families and clinicians exchange in discussing and planning follow-up care.

Secondly, the patients that participated were recovering from recent major surgery and had limited energy for in-depth interviews. The duration of the interviews was adapted to their condition and interviews were bedside held. Sometimes other patients were present in the room. Although these circumstances reflected the real world character of a surgical ward, they felt suboptimal from the researcher’s point of view. However, it did not seem to withhold participants from openness and straightforwardness, resulting in a rich supply of data.

## Conclusion

To improve transitional care decisions for older hospital patients, it is important to understand the needs underlying their preferences. In this study, we identified safety, familiarity, independence, continuity and relief as key needs for older surgical patients. These concepts might be used as a framework to help nurses, physicians and therapists to gain a more comprehensive understanding of their considerations regarding discharge and follow-up care. While other subgroups of older hospital patients may have different needs, the principle of exploring these underlying needs may be applied universally across all older hospital patient populations. We therefore recommend further exploration of needs as an important step toward personalized decision-making in transitional care of older hospital patients.

## Key contributions

**What is already known?**

• About a third of older hospital patients needs follow-up home care or inpatient recovery care.
• To improve transitional care, interventions are recommended that start well before discharge and include communication with the family.
• Follow-up care decisions are tough decisions for older patients.

**What does this study add?**

• Older surgical patients’ needs regarding follow-up care can be captured by five themes: safety, familiarity, independence, continuity and relief.
• Inviting older surgical patients to discuss these five potential underlying needs can help nurses, physicians and therapists to get a comprehensive picture of patients’ considerations regarding discharge and follow-up.
• Gaining insight in the underlying needs of older hospital patients will enhance understanding of their individual preferences for follow-up care and supports the engagement of older patients and families in decision making.

## Declarations

This research did not receive any specific grant from funding agencies in the public, commercial, or not-for-profit sectors.

## CRediT authorship contribution statement

**Aafke J de Groot:** Writing – original draft, Formal analysis, Writing – review & editing, Data curation, Investigation, Conceptualization. **Marike E de Boer:** Writing – review & editing, Methodology, Conceptualization, Validation, Formal analysis. **Elizabeth M Wattel:** Methodology, Writing – review & editing, Conceptualization. **Cees MPM Hertogh:** Supervision, Conceptualization, Writing – review & editing, Methodology. **Marja FIA Depla:** Writing – review & editing, Methodology, Conceptualization, Supervision, Formal analysis.

## Declaration of competing interest

The authors declare that they have no known competing financial interests or personal relationships that could have appeared to influence the work reported in this paper.

## Appendix Supplementary materials

Image, application 1

Image, application 2

Image, application 3

Image, application 4
