# Supplementary material for: Older surgical patients’ preferences for follow-up care after hospital discharge: A multi-method qualitative study into their underlying needs
Source: Int J Nurs Stud Adv. 2025 Jul 29;9:100394. doi: 10.1016/j.ijnsa.2025.100394 (PMC12341641; doi:10.1016/j.ijnsa.2025.100394)
Supplement: Supplementary file 1 [file mmc1.docx]

Supplemental files A. Topic list of patient interviews.

| 1. Introduction |
| --- |
| How are you today? |
| Please tell me about your situation at home. (What kind of house do you live in? Who do you live with?) |
| 2. Hospital stay |
| Why were you admitted to hospital? |
| When was this? |
| Can you tell me what happened since? |
| 3. Discharge |
| Has discharge from hospital been discussed with you? In what way? Who spoke with you about this? When was this? |
| Are you ready to leave hospital? |
| Do you want to go directly home or do you prefer to go to a nursing facility to recover. (Can you tell me some more about that? What do you expect about your recovery?) |
| Did you speak about discharge from hospital with your family? (coming home or going to a facility?) What did they say about it? |
| What did the team tell you about hospital discharge? What did they think about your view? (Could you tell them what you want?) |
| How do you think you will feel the days and weeks after leaving the hospital? What do you look forward to, what do you worry about? How will you deal with this? |
| When date of discharge and discharge destination are decided: What do you think of the arrangements that are made? |
| 4. Review |
| Is there anything else you would like to add? About your discharge and the follow-up care arrangements or how they were made? Or about this interview? |
