# Supplementary material for: Older surgical patients’ preferences for follow-up care after hospital discharge: A multi-method qualitative study into their underlying needs
Source: Int J Nurs Stud Adv. 2025 Jul 29;9:100394. doi: 10.1016/j.ijnsa.2025.100394 (PMC12341641; doi:10.1016/j.ijnsa.2025.100394)
Supplement: Supplementary file 2 [file mmc2.docx]

Supplemental files B. Overview of data collection in each case.

|  | Interview with participant | Informal conversation with family member | Informal  conversation with professional^1^ | Observation bedside visit^2^ | Observation medical rounds^2^ | Report in medical record^3^ |
| --- | --- | --- | --- | --- | --- | --- |
| P1 | x |  | x | x |  | x |
| P2 | x |  |  |  |  | x |
| P3 | x |  |  | x | x | x |
| P4 | x | x | x |  |  | x |
| P5 | x |  | x |  | x | x |
| P6 | x |  |  |  | x | x |
| P7 | x |  |  |  |  | x |
| P8 | x |  | x |  | x | x |
| P9 | x | x |  |  |  |  |
| P10 | x |  |  |  | x |  |
| P11 | x | x |  |  |  | x |
| P12 | x |  |  |  | x | x |

1.Nurse, physician, allied health professional. 2. Nurse, physician. 3. Nurse, physician, allied healthcare professional, medical consultant.
