# Supplementary material for: Older surgical patients’ preferences for follow-up care after hospital discharge: A multi-method qualitative study into their underlying needs
Source: Int J Nurs Stud Adv. 2025 Jul 29;9:100394. doi: 10.1016/j.ijnsa.2025.100394 (PMC12341641; doi:10.1016/j.ijnsa.2025.100394)
Supplement: Supplementary file 3 [file mmc3.docx]

Supplemental files C. Codes, themes and main themes emerging from the individual case histories.

Two examples of a case-specific memo:

| **Codes** | **Themes** | **Main themes** |
| --- | --- | --- |
| Nursing facility for vacuum assisted treatment (VAC)  Knowing what to expect after discharge  Start training  Go home when medical treatment is clear.  Improving one’s condition  Go home when it is safe  Go home after recovery care (in a facility)  Not yet thinking about going home  Worried about wound care in nursing facility  Nursing home for physiotherapy and diet  Family agrees with need for nursing home stay. | Health | 1. Safety |
| Not go back to nursing home care  Trusting nursing home care  Worried about outpatient treatment  Anxious about new setting  Anxious to be alone at night  Can’t go home yet  Worried about nursing home care | Security |  |
| Building up slowly  Anxious and uneasy about recovery  Afraid to start training  Uneasy to move forward in recovery process  Going home is too early  Not being brave now | Prudence |  |
| Preferring a care facility in the neighborhood  Homesick  With my family I can manage.  Trust in support of my own family | Familiarity | 2. Familiarity |
| To be on my own again.  The urge to be out of the hospital  To live independently with my partner  Not becoming old and in need of help  Rather be dead than in a nursing home  Uneasy about slow recovery  Want to be out of the hospital  Push through to recover | Independence/ freedom | 3. Independence |
| I want to be back in my old life  Healthy and taking care of myself again.  Anxious about partner with dementia  Longing for lost capacities  Not wanting to go to a nursing home | Continuity | 4. Continuity |
| Worried that partner/family must handle too much  Worried about pets  Anticipating return home  Regaining strength and abilities  Family can’t do without me | Responsability |  |
| Sudden transfer to rehabilitation facility  Complying to be transferred to a nursing home  Rehabilitation is unknown to us. | Compliance | 5. Relief |
| It has been enough now  I am done with it | Burden of treatment |  |

P3: The patient is naturally strong and optimistic, but now desires a period of recovery. She had the option to either go home or stay temporarily at [nursing home in her neighbourhood], and chose the latter. A significant factor in her decision seems to be the need for regular changes of the VAC pump. ("She also wonders if she might prefer to go home, but the VAC pump, which needs to be changed every three days and is very painful, makes her prefer to stay a little longer for care.") It is also possible that her (foster) son’s reluctance for her to go home influenced her choice.

P11: The patient is very tired and dreads being alone at night if she were to go directly home with home care. She recalls a similar situation 11 years ago. She attributes her fatigue to multiple factors (long waiting times for surgery, having been operated on twice, and being 10 years older). It was later discovered that her haemoglobin was so low that she required a blood transfusion. This does not mean, however, that she is eager to go to a nursing home. She fears that the care there might not be adequate. However, going home with home care is not something she can manage, no matter how much she would like to (even though she wants to care for the dogs when her partner leaves for Spain).
